# Origins and Evolution of the Primate Hepatitis B Virus

**DOI:** 10.3389/fmicb.2021.653684

**Published:** 2021-05-24

**Authors:** Stephen A. Locarnini, Margaret Littlejohn, Lilly K. W. Yuen

**Affiliations:** Victorian Infectious Diseases Reference Laboratory, Royal Melbourne Hospital, The Peter Doherty Institute for Infection and Immunity, Melbourne, VIC, Australia

**Keywords:** hepatitis B virus, genotype, evolution, human migration, ancient DNA

## Abstract

Recent interest in the origins and subsequent evolution of the hepatitis B virus (HBV) has strengthened with the discovery of ancient HBV sequences in fossilized remains of humans dating back to the Neolithic period around 7,000 years ago. Metagenomic analysis identified a number of African non-human primate HBV sequences in the oldest samples collected, indicating that human HBV may have at some stage, evolved in Africa following zoonotic transmissions from higher primates. Ancestral genotype A and D isolates were also discovered from the Bronze Age, not in Africa but rather Eurasia, implying a more complex evolutionary and migratory history for HBV than previously recognized. Most full-length ancient HBV sequences exhibited features of inter genotypic recombination, confirming the importance of recombination and the mutation rate of the error-prone viral replicase as drivers for successful HBV evolution. A model for the origin and evolution of HBV is proposed, which includes multiple cross-species transmissions and favors subsequent recombination events that result in a pathogen and can successfully transmit and cause persistent infection in the primate host.

## Introduction

Infection of the human host with the hepatitis B virus (HBV) can result in a diverse spectrum of clinical outcomes ranging from asymptomatic hepatitis through to cirrhotic liver disease and hepatocellular carcinoma (HCC). Hepatitis B remains a major public health challenge with over 257 million people worldwide presently chronically infected, of whom more than 880,000 persons will die directly each year (World Health Organisation, 2017). Chronic hepatitis B causes almost 40% of cases of HCC and is the second leading cause of cancer-related mortality globally (Stanaway et al., 2016). Long-term outcomes of chronic hepatitis B can vary widely but viral biomarkers, such as HBV genotype and signature mutation profiles in the HBV genome, and serological biomarkers, such as viral load and quantitative levels of Hepatitis B surface antigen (HBsAg) and Hepatitis B e antigen (HBeAg), can predict eventual clinical outcomes (Chen et al., 2007; Yuen et al., 2008; Kramvis, 2014). In terms of host factors, progression to chronic infection is inversely related to age at the time of infection (McMahon et al., 1985; Hyams, 1995), but the final outcome for the exposed individual is dependent on the interaction between host and virus. The more important viral factors include viral genetic variation, viral genotype, and HBeAg status.

In this article, the authors review current theories for the origins of the primate hepadnaviruses putting them in the context of several recent discoveries including advances in recovering HBV DNA sequences from ancient human skeletal and mummified remains (Kahila Bar-Gal et al., 2012; Krause-Kyora et al., 2018; Muhlemann et al., 2018; Patterson Ross et al., 2018) as well as evolutionary processes considered to be involved in the emergence of the major modern HBV genotypes. Finally, we propose a model of HBV origins and diversity drawing on the importance of cross-species transmission and subsequent recombination events not only for the recently discovered ancient HBV (aHBV) strains but also the more contemporary isolates of the virus.

## The Family Hepadnaviruses

### General Considerations

**Figure 1:**
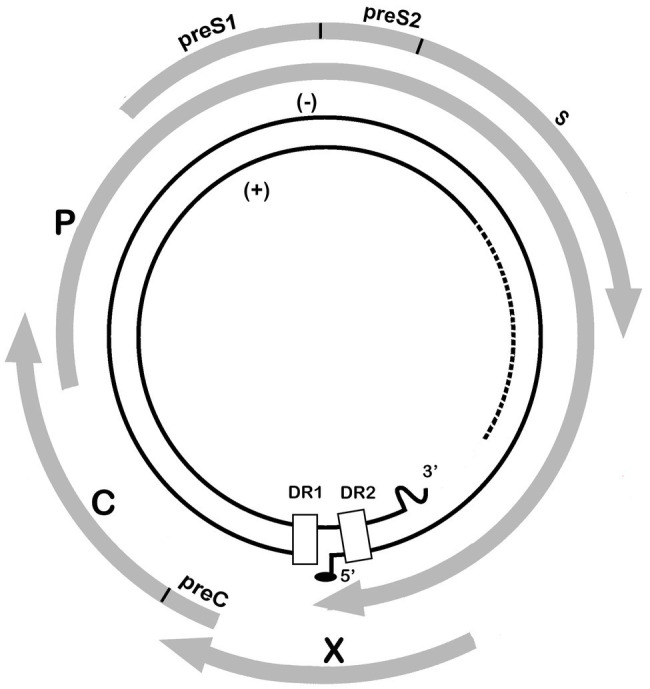
Schematic representation of the HBV genome. The inner circles represent the partial double-stranded DNA molecule and location of the direct repeat (DR) sequences, while the solid gray arrows illustrate the four open reading frames in the HBV genome: polymerase (P), precore (preC), core (C), hepatitis b X protein (X), and envelope (preS1, preS2, and S).

**Figure 2:**
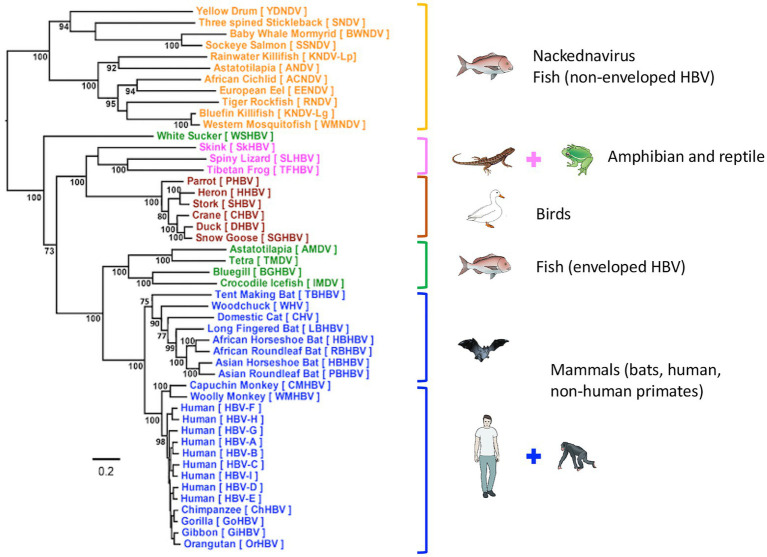
Phylogenetic tree of *Hepadnaviridae*. Phylogenetic analysis of representative hepadnavirus full-length genome sequences from each of the host species known to harbor hepatitis viruses including the HBV genotypes from humans. In general, all HBVs from the same host species cluster together with strong bootstrap support (>80%; with the exception of WSHBV, an enveloped HBV from fish). Based on the available HBV genome sequences, the enveloped and non-enveloped HBVs had distinct evolutionary histories following divergence from their most recent common ancestor (MRCA). The enveloped HBVs are further divided into two major clusters, mammals and fish and amphibians, reptiles, and birds sharing another MRCA. Of these genome sequences, 34 were sourced from GenBank and 14 were annotated sequences from the supplementary data files of Lauber et al. (2017), representing new isolates. The maximum-likelihood (ML) phylogenetic tree was estimated using IQ-TREE v1.6.5 (Nguyen et al., 2015), which is packaged with ModelFinder (Kalyaanamoorthy et al., 2017) and UFBoot (Hoang et al., 2018). Clade support was assessed using 1,000 pseudo replicates generated with the UFBoot non-parametric bootstrap procedure. Branch lengths are scaled to the number of nucleotide substitutions per sequence site. (From (Revill et al., 2020); modified, with permission from the author).

Human HBV is the prototype member of the family *Hepadnaviridae*, which presently includes five genera: the *Orthohepadnavirus* genus that infects mammals, the *Avihepadnavirus* genus that infects birds, the *Parahepadnavirus* and *Metahepadnavirus* genera, which infect teleost fish, and the *Herpetohepadnavirus* genus whose members infect reptiles and frogs (Magnius et al., 2020). All members have similar ultra-structural and molecular genomic features including virion size and morphology, and an enveloped nucleocapsid, which contains a relaxed circular double-stranded DNA genome of 3.0–3.4 kb. This DNA genome is replicated *via* a process of reverse transcription of the key intermediate pregenomic RNA in hepatocytes (Summers and Mason, 1982; Tiollais et al., 1985). Another important feature of hepadnaviral replication is the organization of the DNA into a minichromosome in the nucleus of infected hepatocytes (Bock et al., 1994; Newbold et al., 1995). The HBV genome is organized into four overlapping, but frame-shifted open reading frames (Figure 1). Hepadnaviruses infecting other hosts have recently been identified including bats frogs, lizards, fish, and the capuchin monkey (MacDonald et al., 2000; Drexler et al., 2013; Lauber et al., 2017; de Carvalho Dominguez Souza et al., 2018). The phylogenetic relatedness and relationships within the Hepadnaviridae are shown in Figure 2, with the emphasis of this review on the primate hepadnaviruses, human and non-human.

### Mutation Rate of the Hepadnavirus Genome

Varying interpretations of phylogenetic data have led to uncertainty about the evolutionary history of HBV in humans and non-human primates (NHP; Fares and Holmes, 2002; Starkman et al., 2003) including variable estimates of mutational rates. To some extent, this has been attributed to the anatomy of the HBV genome with overlapping reading frames and the underlying RNA structures embedded within the genome (e.g., epsilon), influencing the evolution of particular regions (Mizokami et al., 1997; Simmonds and Smith, 1999). Estimates obtained for the substitution rate inferred by comparing mother-to-baby transmission infections indicated lower substitution rates in the HBeAg-positive phase (anti-HBe-negative) than in the HBeAg-negative phase (Hannoun et al., 2000; Harrison et al., 2011) by around one order of magnitude. This variance in substitution rates between HBeAg-positive and HBeAg-negative chronic hepatitis B was confirmed by Tedder et al. (2013). The “red queen” hypothesis proposed by these investigators shows that many of the mutations observed in HBV genomes do not generate variability but are reversions back to the genotype consensus (Tedder et al., 2013). The authors concluded that HBV probably behaves as a self-normalizing meme *in vivo* and most of the mutations do not lead to significant changes over time in persistent infection.

Complementing the “red queen hypothesis”, there is increasing evidence that short-time scale studies can artificially inflate evolutionary rates (Li et al., 2017) and the viral genetic diversity rate may decrease over longer time scales; this is referred to as the time-dependent rate phenomenon (Ho et al., 2011; Aiewsakun and Katzourakis, 2016). This reduction rate was found to be consistent with a power-law relationship between substitution rate and observational period (Aiewsakun and Katzourakis, 2017), and all recently identified aHBV fitted remarkably well within this relationship (Simmonds et al., 2019).

### Primate Hepadnaviruses: Modern Genotypes and Subgenotypes

**Figure 3:**
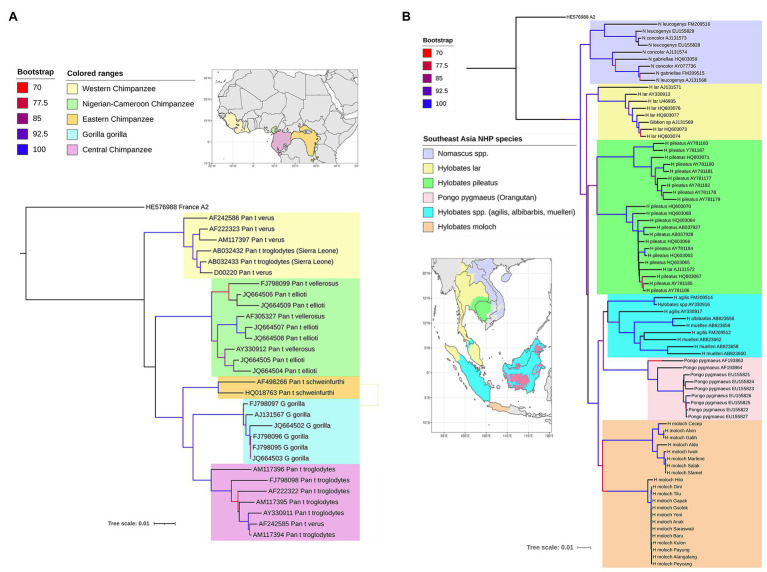
Maximum likelihood (ML) phylogenetic tree of full-length HBV genome sequences extracted from non-human primates **(A)** in Africa and **(B)** Southeast Asia, and reference HBV subgenotype sequences obtained from GenBank. The natural habitat locations of the non-human primates are shown in the corresponding maps, with same the color code used on the ML trees for the different host species. The trees were inferred using IQ-TREE v1.6.5 (Nguyen et al., 2015). The best-fit models determined by ModelFinder (Kalyaanamoorthy et al., 2017) were GTR+F+R2 and GTR+F+R4, respectively. Clade support was assessed using 1,000 pseudo replicates generated with the UFBoot non-parametric bootstrap procedure (Hoang et al., 2018), and bootstrap values >70% are shown by a color scale on the tree branches. The ML tree was annotated using iTOL v5.7 (Letunic and Bork, 2019). Branch lengths are scaled to the number of nucleotide substitutions per sequence site.

The primate hepadnaviruses are indigenous to their hosts (Norder et al., 1996; Lanford et al., 1998; Warren et al., 1999; Grethe et al., 2000; Robertson and Margolis, 2002; Sall et al., 2005), and the phylogenetic analysis of full-length genomes from the different primate HBVs, both human and non-human, reveals that they essentially cluster according to the geographical locations of host habitats, particularly for NHP HBV (Starkman et al., 2003; Kramvis, 2014). For example, HBV from central chimpanzees (*Pan troglodytes*) are genetically more related to HBV collected from gorillas, with whom they share an overlapping geographical range (the region south of the Sanga River in Cameroon and west of the Oubangui River in Congo, Zaire; Figure 3A; Hu et al., 2001), than to HBV from other common chimpanzee subspecies in Africa (Figure 3A). Likewise, HBV isolated from central, eastern (*Pan troglodytes schweinfurthii*), and Nigerian-Cameroon (*Pan troglodytes ellioti* and *vellerosus*) chimpanzees, with adjacent habitats, formed sister clades. While HBV sequences from western chimpanzee (*Pan troglodytes verus*, west of the Niger River) formed the most distal clade of all African NHP HBV, and their habitat is the most distant from the other common chimpanzees (Figure 3A). Surprisingly, no HBV isolates have been identified from the bonobo (*Pan paniscus*) chimpanzee. The same observations are seen with the Southeast Asian NHP, where the orangutan HBV sequences cluster more closely with those from Bornean gibbons (*Hylobatidae muelleri* and *Hylobatidae albibarbis*) and Island Southeast Asian gibbon (*Hylobatidae agilis*; Figure 3B). The other gibbon species from mainland areas of Southeast Asia form a separate group.

The phylogenetic relationships of the NHP HBV are reflected in the nucleotide divergence data. The highest divergence is found between the African and Asian NHP HBVs, which diverge by 10–11% at the nucleotide level. Within the African groups, the divergence between the various chimpanzee and gorilla HBV species is 5–7%, while within the Asian species, orangutan, and gibbon HBV species, the divergence is slightly higher at 7–9%. The gibbon HBV sequences can be further split into mainland species, where the divergence is 7–8%, compared to the island species, where the divergence is slightly higher at 8–9%. Using the accepted definition for human HBV genotypes of >7.5% divergence, each of these NHP HBV species is regarded as individual genotypes.

**Figure 4:**
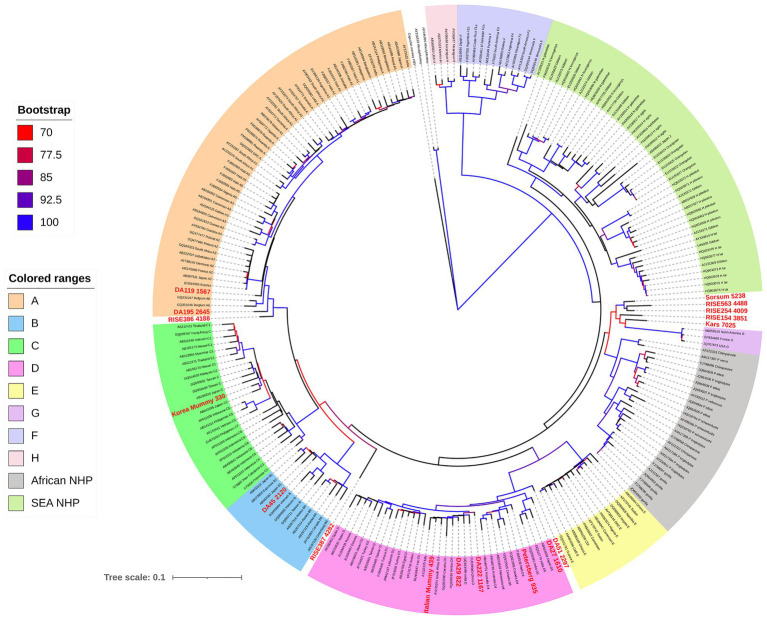
Maximum likelihood (ML) phylogenetic tree of full-length HBV genome sequences (aHBV has shown in bold red font) extracted from human and non-human primate hosts, and reference HBV subgenotype sequences obtained from GenBank. For aHBV, the number after ID represents the approximate age of the source fossil samples. The tree was inferred using IQ-TREE v1.6.5 (Nguyen et al., 2015), which is packaged with ModelFinder (Kalyaanamoorthy et al., 2017) and UFBoot (Hoang et al., 2018). The best-fit model determined by ModelFinder was GTR+F+R5. Clade support was assessed using 1,000 pseudo replicates generated with the UFBoot non-parametric bootstrap procedure, and bootstrap values >70% are shown by a color scale on the tree branches. The ML tree was annotated using iTOL v5.7 (Letunic and Bork, 2019). Branch lengths are scaled to the number of nucleotide substitutions per sequence site.

Phylogenetic analysis, including both human and NHP HBV, shows both groups form unique clades, separate but interspersed (Figure 4; Locarnini et al., 2013). The nucleotide divergence between human and Old World NHP HBVs range 10–15%, but is substantially higher when compared to New World NHP HBVs. The level of nucleotide divergence between HBV from Woolly Monkey and Capuchin Monkey, when compared to all other primate HBV sequences are 28% (Lanford et al., 1998) and 20% (de Carvalho Dominguez Souza et al., 2018), respectively.

It is worth noting that NHP HBV has several distinctive amino acid replacement features compared to a consensus sequence derived from each of the human genotypes (Robertson and Margolis, 2002), and some of these features were also detected in the NHP-like aHBV (Table 1). The NHP HBV distinctive amino acid replacement features include:

1. Pre-S region: a glutamic acid (E) at position 16 in chimpanzee, gorilla, and gibbon isolates.
2. S-region: three amino acid changes (L133, I/L/Y134, and A177) in the chimpanzee, gorilla, and gibbon HBV isolates.
3. Core antigen: the typical precore stop codon at position 28 (associated with HBeAg negativity) has a leucine (L) at this position in contrast to tryptophan (W) in all the human isolates. Likewise, in the core region itself, glutamine (Q) at position 113 of the chimpanzee and gorilla HBVs replaces a leucine (L) in human HBV. In addition, Takahashi et al. (2000) highlighted a proline (P) alanine (A) motif within five amino acids from the termination of the core protein (C-terminal) found in all NHP HBV sequences as well as human genotypes E and F/H and genotype G (Stuyver et al., 2000).
4. Pol region: an 11 amino acid deletion in the pol gene and the N-terminal portion of the Pre-S1, which is shared with the human genotype D strains.
5. X-gene: three amino acid changes (T11, K107, and T110) in chimpanzees and two (K107 and T110) found in gorilla sequences.

**Table 1:** Key distinctive amino acid replacement features in non-human primate HBV sequences of aHBV compared to sequences derived from human genotypes.

Host species or Ancient isolate ID	Archeological Period	Approximate sample age (years)	Genotype	PreS1 deletion (Pol overlap)	Precore amino acid position 28	Precore amino acid position 2	Core amino acid position 113	“PA” motif at 3' end of Core
Human HBV	Modern	-	A–J	Only in Geno D	W/*	Q/*	Mostly “L”	RESQC* except Geno E, F/H, G
NHP* HBV	Modern	-	NHP	Present	Mostly L	Q	“Q” in African “L” in Southeast Asian	PASQC*
Korean Mummy	Early modern	330	C2	Undetermined	W	Q		Undetermined
Italian Mummy	Early modern	439	D3	Present	W	Q		RESQC*
DA29	Medieval	822	D3	Present	W	Q/*	L	RESQC*
Petersberg	Medieval	935	D4	Undetermined	W	Q	L	RESQC*
DA222	Medieval	1,167	D3	Present	W	Q	L	RESQC*
DA119	Medieval	1,567	A	Absent	W	Q	L	RESQC*
DA27	Iron	1,610	D5	Present	W	Q	L	RESQC*
DA45	Iron	2,120	B1	Absent	W	Q	L	RESQC*
DA51	Iron	2,297	D	Present	W	Q	L	RESQC*
DA195	Bronze	2,645	A	Absent	W	Q	L	RESQC*
RISE386	Bronze	4,188	A (ancient)	Absent	W	Q	L	RESQC*
RISE387	Bronze	4,282	A (ancient)	Absent	W/*	*	L	PASQC*
RISE154	Bronze	3,851	NHP (extinct)	Undetermined	L	Q	Q	PASQC*
RISE254	Bronze	4,009	NHP (extinct)	Present	L	Q	Q	PASQC*
RISE563	Bronze	4,488	NHP (extinct)	Present	L	*	Q	PASQC*
Sorsum	Neolithic	5,238	NHP (extinct)	Undetermined	L	Q	Q	PASQC*
Karsdorf	Neolithic	7,025	NHP (extinct)	Undetermined	L	*	Q	PASQC*

*indicates stop codon.

Interestingly, the Pre-S1 deletion, the precore amino acid at position 28, the core amino acid position at L113Q, and the “PA” motif at the 3' end of core are also identified as “finger-print” differences in the NHP aHBV. The distinctive amino acid motifs in the S-region and core antigen in NHP compared to human sequences may be a result of species-specific responses to host immune pressure; both the surface and core antigens are highly immunogenic. These motifs could also be potentially a result of convergent evolution as they appear in both the African and Asian NHP HBV lineages, which are separated by human HBV genotypes. The modification at precore position 28 could be associated with a lack of disease in NHP, as a mutation at this position results in complete abrogation of expression of the HBeAg. The HBeAg negative phase in human genotypes is associated with liver disease progression, which does not generally occur in primate HBV infection and so these substitutions could be acting as compensatory changes [see section The Extinct Neolithic Genotype (5–7 kya) and Table 1]. Finally, the 11 amino acid deletion in the Pre-S1 gene and overlapping polymerase region could possibly have some effect on species-specific viral entry, as this is adjacent to the essential NTCP binding element on the viral surface gene.

**Figure 5:**
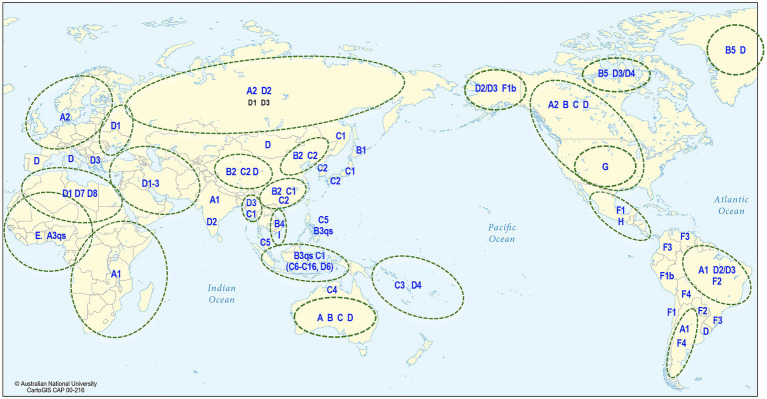
Global distribution of HBV. The map shows the predominant genotype and subgenotypes that have been identified in different countries around the world.

Human HBV is currently grouped into nine genotypes (designated A–I) and one putative genotype J, based on a full genome diversity of more than 7.5% at the nucleotide (nt) level (Okamoto et al., 1988; Norder et al., 1994; Tran et al., 2008; Tatematsu et al., 2009; Kramvis, 2014), and the phylogenetic analyses of aligned full genome sequences are shown in Figure 4 (Miyakawa and Mizokami, 2003; Norder et al., 2004; Olinger et al., 2008). HBV genotypes are geographically distributed across the globe (Figure 5). Generally, Generally, HBV genotypes are associated with distinct clinical outcomes ranging from mild hepatic disease (subgenotypes B1 and B5) to rapid progression to liver failure and malignancy (genotypes F and C). Also affected by genotype are HBeAg seroconversion rates and the emergence of mutation profiles in the basal core promoter and precore regions of the viral genome associated with HBeAg loss (Rodriguez-Frias et al., 1995; Chu et al., 2003; Liaw and Chu, 2009).

Phylogenetic analyses have shown that most of the genotypes have sufficient diversity to be categorized into subgenotypes differing by at least 4% (Kramvis and Kew, 2005) of their full genome sequence. Genotypes E, G, and the putative J are not subdivided into subgenotypes and this might reflect idiosyncratic or uniquely different epidemiological patterns such as a more recent origin or re-introduction following a relatively recent extinction or loss. The distribution of HBV subgenotypes is also generally geographical (Figure 5). For example, subgenotype A1 is found mainly in Africa, Asia, and Latin America with dispersal outside Africa suggested to have occurred as a result of the slave trade (Kramvis and Paraskevis, 2013), while A2 is in Europe and North America (Sugauchi et al., 2004); B1 is found in Japan, especially in the south, while B2, a recombinant with genotype C in the precore-core region, is found in the rest of Asia (Sugauchi et al., 2004); C1 is the dominant strain in South and Southeast Asia, while C2 is found mainly in North Asia especially Korea (Huy et al., 2004; Chan et al., 2005). Subgenotype C4 is found exclusively in Australian Indigenous persons and its envelope protein is possibly regarded as the original “Australia Antigen” (Littlejohn et al., 2014), while C3 is found only in Melanesian populations of the South Pacific. Subgenotypes C3 and C4 most probably diverged from ancestors of subgenotypes C1 and C2 prior to being carried south and east to Melanesia and Australia (Yuen et al., 2019). Subgenotypes D1–D4 are widely distributed globally (Kimbi et al., 2004; Banerjee et al., 2006), with D3 common in Europe and the Mediterranean region while D4 is the dominant subgenotype in Polynesia and Micronesia (Jackson et al., 2020), and also found in India and the Arctic Denes (Banerjee et al., 2006; Osiowy et al., 2011).

## Ancient HBV Isolates

Over the last decade, technological advances have allowed larger-scale analysis of next-generation sequencing data (Qin et al., 2010) including multiple studies utilizing fossilized and mummified tissue and skeletal remains for metagenomics composition. This has provided deeper insights into the identification of diseases from which that particular individual suffered (Cho and Blaser, 2012; Lynch and Pedersen, 2016). Although the analysis can be limited by variations in sample preservation as well as the presence of environmental contamination, studies have obtained well-preserved microbial, viral, and human DNA especially from the dental calculus (Preus et al., 2011; Warinner et al., 2014; Ozga et al., 2016). This has enabled the simultaneous study of pathogen and host. Mummified remains have also presented an opportunity to detect specific pathogens and perform human and microbial genome reconstruction, when combined with genome-wide DNA capture approaches (Bos et al., 2011) such as the identification of an ancient *Helicobacter pylori* genome from the 5,000-year-old Tyrolean Iceman (Maixner et al., 2016).

For aHBV, the oldest reconstructed genomes have been dated to the Neolithic, 5,000–7,000 years ago (kya; Krause-Kyora et al., 2018) and the Bronze Age 3–4 kya (Muhlemann et al., 2018) using dental and skeletal remains. It is likely that these represent exogenous HBV genomes rather than HBV integrated into the human genome based on the fact that the samples were extracted from teeth. aHBV has also been detected in two preserved mummies, one from Korea and the other from Italy (Kahila Bar-Gal et al., 2012; Patterson Ross et al., 2018) dated from around 400 years ago (ya). Phylogenetic reconstruction of HBV isolated from these samples revealed that the oldest HBV genomes, which were from the Neolithic period and appear extinct, are most closely related to the HBV of African NHP (Table 1; Krause-Kyora et al., 2018; Muhlemann et al., 2018). The oldest classifiable genotype was aHBV-A found in Russia dated around 4,200 years ago. For further comparisons, the aHBV from these two studies as well as the isolates identified from the Italian and Korean mummies can be grouped under the following time-frame categories: (a) Extinct Neolithic Genotype, (b) Bronze Age Genotype, (c) Medieval Genotype, and (d) Early Modern Genotype (Table 1).

### The Extinct Neolithic Genotype (5–7 Kya)

Two isolates were identified from fossils across a narrow geographical range in Western Europe mainly in present-day Germany at Karsdorf (7 kya) and Sorsum (5.2 kya). Although the age of these two strains were estimated to be 2,000 years apart, they showed a higher genomic similarity (6%) to each other and African NHP HBV (<8%) than to any modern human HBV (Figure 4; Krause-Kyora et al., 2018). The authors identified some fragments of the two aHBV genomes showing high similarity to human HBV genotypes E and G and proposed that the aHBV from Karsdorf may have been a distant source for the younger Sorsum virus. Given the genetic distances of these aHBV were <8% from African NHP HBV they were not classified as a novel genotype. Nonetheless, they could be classified as an extinct subgenotype of HBV, given they are no longer found circulating in humans. Both these sequences displayed the sequence motifs detected in modern NHP. Although the Karsdorf sequence has a leucine at precore position 28, it also has the BCP mutations at 1762/1764 as well as a precore stop codon (PC_Q*2), which would abrogate expression of the viral HBeAg.

### Bronze Age Genotype (Approx. 2–4 Kya)

Isolates RISE563 (Germany), RISE254 (Hungary), and RISE154 (Poland) identified from an independent study (Muhlemann et al., 2018) were also found to be genetically related to the Neolithic aHBV discussed above and are closely related to the African NHP HBV with the corresponding sequence motifs although the RISE563 sequence has a stop codon in the precore gene (PC_Q*2; Table 1; Figure 4). Given European great apes have been extinct since the late Miocene (at least 7 million years ago (mya); DeMiguel et al., 2014), the presence of HBV most closely related to African NHP HBV in these locations suggest at some stage in the evolutionary history, a possible African origin for these extinct aHBV genotypes.

Phylogenetic testing of the Bronze Age isolates showed the RISE386/387 samples, which were collected from Bulanovo Russia, had clustered as basal partners within clade genotype A. Interestingly, the RISE387 has some, but not all NHP sequence motifs, while the RISE 386 has predominantly human HBV sequence motifs (Table 1). Although the isolate RISE387 does not consistently cluster with genotype A as shown by Datta (2020), it is <8% genetically different from contemporary genotype A isolates. This suggests the geographical origin of genotype A may have been Central Asia, in agreement with studies using contemporary sequences, which also concluded a Middle East/Central Asia origin for genotype A (Kramvis and Paraskevis, 2013; Paraskevis et al., 2013; Kostaki et al., 2018). In this scenario, Muhlemann et al. proposed that the ancestors of subgenotype A1 and quasi-subgenotype A3 would have been carried into Africa following migration from Eurasia (Muhlemann et al., 2018). In addition, the oldest isolate of genotype D (DA51) was identified from a 2,000-year-old Iron Age fossil, and it was considered by these investigators as the ancestral genotype D (Muhlemann et al., 2018). This aHBV was collected from a fossil in Kyrgyzstan, Central Asia, and has all human HBV sequence motifs. Also of interest from this study was the identification of the isolate DA45 that was classified as subgenotype B1 from Mongolia, East Asia, indicating that this non-recombinant subgenotype may have originated in other parts of East Asia before becoming the dominant genotype B in Japan. This finding also indicates that the Asian dominant strains like genotype B had already established their geographical niche over 2 kya.

### Medieval Genotype (Approx. 1 Kya)

Both the Krause-Kyora et al. (2018) and Muhlemann et al. (2018) studies identified HBV genomes from fossil samples dated around 1,000 years old. These included a HBV-D4 from Petersburg, Russia, and two HBV-D3 from Kazakhstan in Central Asia (DA29 and DA222). Of interest, the two isolates that were over 1,000-year old clustered as basal taxons in the subgenotype clades, while isolate DA29 (829-year old) clustered among the contemporary HBV-D3.

### Early Modern Genotypes

Ancient DNA studies have also provided new perspectives on the more recent evolutionary history of HBV. HBV genomes have been sequenced from a Korean child mummy radiocarbon dated to 330-year BP (±70 years), translating to ca. 1,682 (1,612–1,752; Kahila Bar-Gal et al., 2012) and a 439-year BP (±60 years) Italian mummy from Naples, Italy around the same time frame (Patterson Ross et al., 2018). Phylogenetic analysis of the HBV DNA from the mummified Korean child positioned it within the cluster of modern subgenotype C2 (Figure 4). Likewise, the phylogenetic analysis of HBV sequence from the Italian mummy (Figure 4) revealed that the HBV sequence reads were of subgenotype D3. These phylogenetic results suggest low long-term mutation rates, with genotypes diversifying over many thousands of years (Paraskevis et al., 2013). This finding highlights that the genomic structure of HBV has strong selective constraints as described in section “The Family Hepadnaviruses.” There was no evidence of recombination from either the Korean mummy or Italian mummy HBV genome sequences (Patterson Ross et al., 2018).

## Mixed Co-Infections and Recombination

### Mixed/Co-infection of HBV

The co-existence of different HBV genotypes, resulting from multiple exposure and super-infection, can lead to exchange of genetic material between two viral strains infecting the same cell resulting in a recombination event (Fares and Holmes, 2002; Hannoun et al., 2002). The actual mechanism by which inter-genotype recombination occurs remains unknown but there are multiple occasions throughout the HBV life cycle, where this may occur (Newbold et al., 1995; Yang and Summers, 1995; reviewed in Littlejohn et al., 2016).

### Modern HBV Recombinants

**Figure 6:**
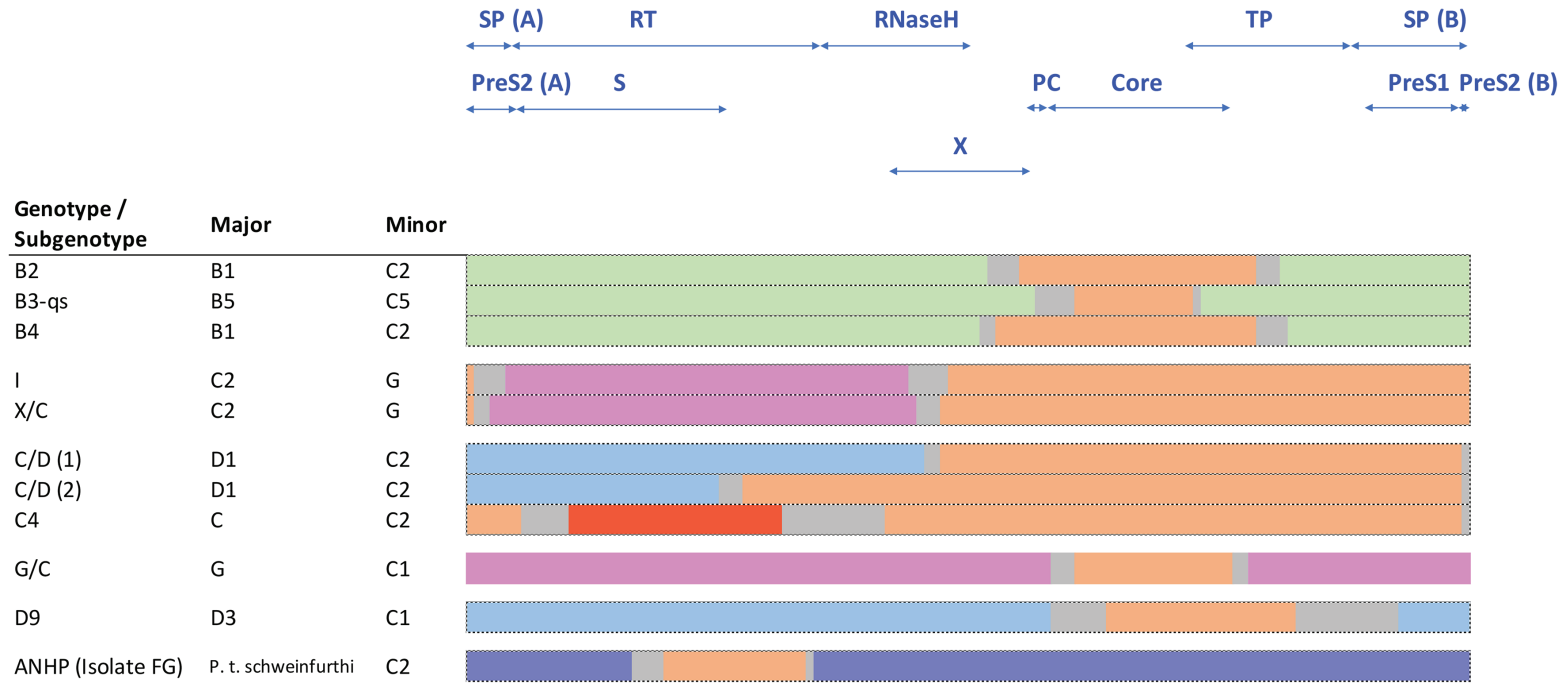
Schematic representation of known HBV genotype C recombinants, showing the conserved regions of the major and minor components. Sequences are color coded by genotype: genotype B (green), C (orange), D (blue), G (pink), J (red), and chimpanzee (purple). The space between the conserved regions is due to uncertainty about the precise recombination breakpoints and is indicated in gray.

Several HBV recombinants have been assigned their own genotype or subgenotype and are endemic in certain geographic regions. Interestingly, the majority of known HBV recombinants involve HBV genotype C either as a major or minor parental sequence (Figure 6). Most notable is the HBV subgenotypes B2-B4, which are recombinants between isolates of HBV genotypes B and C (Sugauchi et al., 2002). These contain the precore and core genes of HBV genotype C and are found throughout Asia (Figure 6; Sugauchi et al., 2002). As highlighted above, the subgenotypes B1 and B5 are not recombinants and are mainly confined to Japan and the circumpolar Arctic, respectively, while HBV genotype I has been reported as a triple recombinant containing elements from genotypes A, G, and C and are predominantly found in Vietnam and Southern China (Tran et al., 2008). Recognized HBV recombinants that localize to specific geographical regions have also been described including the various C/D-recombinants circulating in Tibet (Cui et al., 2002; Wang et al., 2005; Zeng et al., 2005). Other genotype C recombinants are shown in Figure 6.

The overlapping reading frames and viral regulatory elements embedded within coding regions of HBV genomes give rise to alternating regions of variable and conserved domains that can be described as mosaic structures (Bowyer and Sim, 2000). The more variable of these domains have been associated with recombination events rather than the result of point mutation accumulation. Simmonds and Midgley (2005) have also described the HBV genome as modular, representing a blend of small segments from genomes of different human and primate HBV strains. It is possible that genome mosaicism is an important driving force in the evolutionary history of HBV genotypes. Pathogenesis of chronic hepatitis combined with host-virus interaction among different host populations can result in the selection of different combinations of allelic modules with different properties that would improve viral fitness (Littlejohn et al., 2016).

Simmonds and Midgley (2005) developed the TreeOrder scan methodology to enable a large number of DNA sequences to be simultaneously analyzed for potential recombination events, on HBV genomes. Most (90%) of the recombinants detected were either hybrids of genotypes B/C or A/D with A and C showing a higher tendency for recombination than other genotypes. These studies mapped up to eight breakpoint hotspots in HBV genomes, where inter-genotype recombination tend to occur. A comprehensive review on global HBV inter-genotypic recombinants has been published, drawing on over 400 complete HBV sequences (Araujo, 2015), highlighting some of the challenges in studying and developing models for the origins of HBV. Inter-species recombination events between human and chimpanzee HBV have also been described. Studies have characterized an HBV recombinant that was isolated from a wild-caught chimpanzee (*P. t. schweinfurthii*) in East Africa (Vartanian et al., 2002; Figure 6). The segment of human HBV subgenotype C2 detected in the HBV polymerase genome region of this isolate (FG, Figure 6) support the occurrence of recombination among various primate HBV strains. Such recombination events may have played an important role in the current distribution of HBV genotypes and subgenotypes following *Homo sapiens* exit from Africa and subsequent global diaspora. It is interesting to note that Datta, (2020) observed components of chimpanzees and subgenotype C2 HBV in aHBV samples from the Bronze Age (RISE 386/387) that were distantly related to the ancestral genotype A, from around 4 kya isolated from Bulanovo, Russia.

### Ancient HBV Recombinants

From the sequences of aHBV published by Krause-Kyora et al. (2018) dating from the Neolithic period, a number of recombination events were identified. Fragments of the Karsdorf sequences showed high similarity to modern human HBV genotypes G and E as well as NHP HBV sequences. Part of the Sorsum HBV genome showed high similarity to the human genotypes G, E, and B. Likewise, Muhlemann et al. (2018) found strong evidence that aHBV sequence of DA51 (ancestral D) and an unknown parent recombined to form the ancient genotype A sequence. Their predicted recombination break points corresponded closely to the polymerase gene implying that the polymerase from an unknown parent and the remainder of the genome from an HBV DA51 ancestor recombined to form ancestral genotype A about 7.4–9 kya (ancestral A).

An independent analysis by Datta (2020) confirmed and extended these findings and suggested a RISE563-like sequence could have contributed to existing genotype I sequences, which is composed of genotypes A, C, and G. Again, this analysis suggested NHP-like HBV sequences were major contributors to the Neolithic and early Bronze Age aHBV sequences with minor contributions from HBV genotype D (subgenotype D6) and E-like sequences. Furthermore, sequences similar to genotype C (subgenotype C2) and gibbon HBV were detected in Neolithic and Bronze Age aHBV isolates from Europe/central Asia (Datta, 2020). Considering genotype I is a triple recombinant of genotype A, C, and G, these findings may not be that surprising after all.

### The Australia Antigen and HBV-C4

Indigenous Australians have the oldest continuous living culture outside of Africa with studies showing both Melanesian and Australian Indigenous populations have ancient mitochondrial DNA haplotypes (Merriwether et al., 2005). These two major populations of Sahul are associated with infection with unique HBV genotypes, HBV-C4 for Indigenous Australians and HBV-C3 for Indigenous Melanesians. Davies et al. (2013) reported that HBV isolated from Indigenous Australians who reside in the Northern Territory (NT) of Australia are exclusively infected with HBV-C4 and this subgenotype has not been found in any other population. HBV-C4 is a recombinant subgenotype containing a genotype C backbone with a genotype J surface gene (Littlejohn et al., 2014). Given genotype J is most similar to HBV isolated from SEA gibbons and orangutans (Tatematsu et al., 2009) and these primates are only found west of the Wallace Line (Wallace, 1876), the faunal boundary line that separates the eco-zones of Asia and Australia. The presence of the HBV-J surface gene in the HBV-C4 recombinant and the exclusivity of HBV-C4 in Indigenous Australians from the NT suggests the recombination event occurred during the migration of anatomically modern humans (AMH) to Australia approx. 50 kya (Yuen et al., 2019). The HBV-C3 in contrast is not a recombinant virus, but is almost universally associated with the Indigenous Melanesian population of Sahul and the Solomon Islands.

## Primate Origins, Evolution, and Migrations

Given the origin of HBV in primates may be associated with host evolution, it would be reasonable to propose that primate origins, evolution, and migrations may have a role in the evolutionary history of HBV. Nonetheless, further studies are required to allow a definitive conclusion. It is worth noting that co-evolution may be unlikely as the data in Figure 4 shows the relationships between human and NHP HBV do not follow host phylogeny, thus not supporting a co-evolution theory. A proposed timeline in primate and human evolution based on fossil evidence is shown in Table 2.

**Table 2:** The fossil evidence proposes the following timeline in primate and human evolution.

Timeline	Primate and human evolution
45 mya	Primates evolved in Asia and rapidly dispersed and colonized in Africa ^a^^,^^b^^,^^c^
35	Old World and New World monkeys split ^d^
20.4	Gibbons split ^e^
15.7	Orangutans split ^e^
8.8	Gorillas split from the human-bonobo-chimpanzee ancestor ^e^
6.4	Australopithices/Homo ancestors split from the bonobo-chimpanzee lineage ^e^
1.8	*Homo erectus* emerged from Africa and dispersed and colonized China (Peking Man) and Java (Java Man) ^f^
>400 kya	*Homo sapiens neaderthalensis* emerged in the Levant ^g^
>300 kya	*Homo denisova* detected in Siberia/Tibet Plateau ^h^
>200 kya	*Homo sapiens sapiens* emerged in Africa ^i^

a Beard et al. (1994);

b Kay (2012);

c Chaimanee et al. (2012);

d Godinot (2020);

e Kumar and Hedges (2011);

f Zaim et al. (2011) and Zhu et al. (2018);

g Green et al. (2010);

h Reich et al. (2010);

i Bergstrom et al. (2021).

### Asian Primates Origins: Middle Eocene

It is generally held that stem anthropoids arose in Asia 45 mya and that one or several anthropoid groups later migrated to Africa in the late Eocene (Kay, 2012); there is no convincing evidence for the existence of anthropoids in Africa before 38 mya. Specifically, Beard et al. (1994) discovered a fauna of primates from the Eocene dated to around 45 mya from deposits in Shanghuang, Jiangsu Province, China. These fossils included the first Eocene Tarsier as well as fossils subsequently shown to form the basal simians (anthropoids/higher primates), which became known as the Eosimias. These findings established Asia as the biogeographical origin for primate evolution and were subsequently confirmed from fossil discoveries made in Myanmar in 2012 with the identification of *Afrasia djidjidas*, a new form of anthropoid primate that was at least 40 million years old (Chaimanee et al., 2012). Interestingly, this fossil find resembled another early anthropoid, *Afrotaesius lybicus* that had been discovered in the Sahara Desert of Libya (Jaeger et al., 2010) and dental examinations revealed *Afrasia* to be more primitive than *Afrotarsius*, further establishing that early anthropoids originated in Asia first and then quickly colonized Africa. The movement into Africa by these early anthropoids was crucial for subsequent primate and human evolution, with the subsequent emergence of more advanced apes and hominoids there. This ancient migration required the crossing of the Tethys Sea that separated Africa from Eurasia in the late Middle Eocene (Jaeger and Marivaux, 2005). The successful colonization of Africa by Asian anthropoids across the Tethyan marine barrier was possibly by island hopping, similar to that proposed for the arrival of the ancestors of the New World monkeys into the Americas (Table 2).

### New World Monkeys

Two New World monkeys have been found to be infected with their own hepadnaviruses; capuchin monkeys and woolly monkeys. The woolly monkeys comprise the genus *Lagothrix* with four species: *Lagothrix lagotricha*, *L. cana*, *L. lugens*, and *L. poeppigii*. These animals are found throughout the northern humid rainforests of South America in Bolivia, Colombia, Ecuador, Venezuela, and Peru. The woolly monkey species *L. lagotricha* was shown in 1998 to be infected with the woolly monkey HBV (WMHBV) found in captive woolly monkeys housed in the Louisville KY zoo (Lanford et al., 1998). Interestingly, six monkeys had been obtained from a zoo in Scotland in 1985. Inoculation of WMHBV into spider monkeys and chimpanzees resulted in the detection of virus replication in both animals and the development of anti-HBc positivity in the spider monkeys. However, no evidence of raised liver function tests (LFT) was obtained detectable hepatic disease. More recently, Souza and colleagues (de Carvalho Dominguez Souza et al., 2018) sampled multiple sites in Bahia State, Brazil and identified a new HBV in the species *Sapajus* (family Cebidae), the capuchin monkey (or the “organ grinder” monkey). Maximum likelihood phylogenetic analysis of full-length primate HBV genomes revealed that the capuchin monkey HBV (CMHBV) and WMHBV clustered together with high statistical support, forming a basal sister lineage to the HBV genotypes (Figure 4; de Carvalho Dominguez Souza et al., 2018). The high level of nucleotide divergence between these New World NHP HBV and other primate HBV may be associated with an early divergence followed by very long-term isolation, possibly soon after the split between Old and New World monkeys around 35 mya. Importantly, though the modern human HBV genotypes F and H are predominantly found in the Americas, the phylogenetic analysis strongly suggests their origin is in the Old World with no evidence of cross-species transmission with the New World NHP HBV.

A frequently asked question is “How did these New World animals arrive in South America from West Africa?” The order *Simiiformes* split into the Platyrrhini (New World monkeys) and Catarrhini (apes and Old World monkeys) about 33–35 mya in Africa and Eurasia. Furthermore, it has been suggested that the ancestors of the Platyrrhini migrated to South America either on a raft of vegetation or *via* a land bridge (Beard, 2004). Two possible rafting routes have been suggested, either across the Atlantic Ocean from Africa (then the Afro-Arabian plate; Godinot, 2020; Seiffert et al., 2020) or across the Caribbean from North America. The former would have the New World monkey ancestors sail on the south equatorial paleo current (SEC), 35–32 mya during the Oligocene (Godinot, 2020). The land bridge route would need to rely on the existence of Atlantic Ocean ridges and a significant fall in the sea level during the Oligocene. At that time, the Atlantic Ocean was less than its present 2,800 km width, by about one-third, at around 1,000 km or less (Beard, 2004). This may have resulted in either a single land bridge or a series of mid-Atlantic islands that could act as stepping stones.

### African Origins and Out-of-Africa: Again and Again

**Figure 7:**
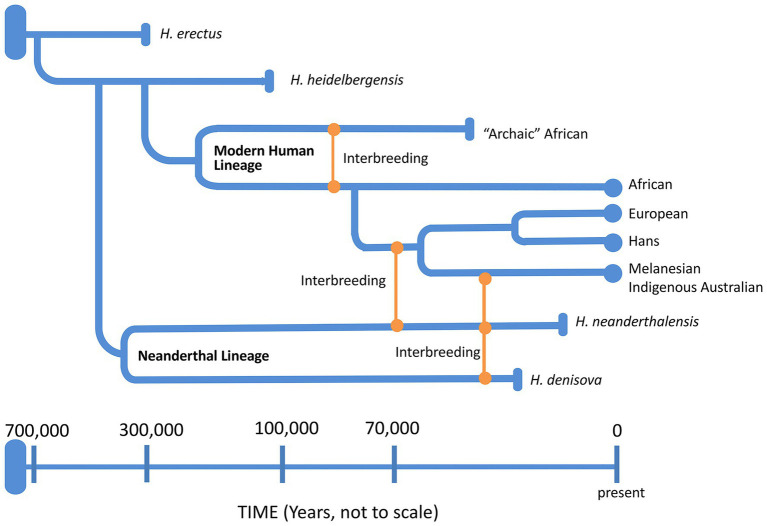
A schematic representation of the genus *Homo* evolution timeline over the last 700,000 years, showing the admixture relationships between *Homo sapiens*, during migration out of Africa, and other archaic humans. At the end of each lineage, circle marks represent modern humans and rod marks represent archaic humans who are now extinct.

Around 6–8 mya, the ancestors of modern humans began to evolve in Africa (Table 2), approximately around the same time that *Australopithices* split from the bonobo-chimpanzee lineage. *Homo sapiens* emerged in Africa around 200 kya while the Neanderthals (*H. sapiens neanderthalensis*) evolved from *H. heidelbergensis* around 400 kya, not in Africa but probably in the Levant (Middle East) or Western Asia region. Neanderthals went extinct around 30 kya with the last evidence for their existence in caves in and around Gibraltar. Complete nuclear genomes of archaic humans have been successfully reconstructed from fossil remains of not only Neanderthals (Green et al., 2010), but also Denisovans from the Denisova cave in Southern Siberia (Reich et al., 2010), and an archaic human who lived in Nigeria (Hammer et al., 2011). All three genomes were more than 35,000 years old with clear genetic evidence of interbreeding among archaic humans on at least three occasions (Reich, 2018). Potentially then, as AMH evolved in and left Africa by around 40 kya (northern dispersal route), they interbred with Neanderthals in the Middle East or Arabia before spreading out into Asia and Europe (Green et al., 2010; Figure 7). Another group of humans had earlier left Africa around 70 kya (Rasmussen et al., 2011; southern dispersal route) and these humans headed east along the coastal route toward Australia and Melanesia interbreeding with the Denisovans on the way (Figure 7). Thus, Indigenous Melanesians inherited DNA from both the Neanderthals and Denisovans during the first wave of expansion into Melanesia, having up to 8% of their DNA coming from these archaic peoples (Reich et al., 2010), which is distinct to the second wave during the Neolithic period (10,000–3,000 BC) when people migrated from East Asia during the Austronesian Expansion to expand into Polynesia (Kirch, 2017). Other populations in Southeast Asia and Oceania have also been found to carry Denisovan admixture, including Indigenous Australians, Polynesians, Fijians, East Indonesians, and Mamanwa but not mainland East Asians, Western Indonesians, Jehai, or Onge (Reich et al., 2011). The successful sequencing of an Indigenous Australian’s 100-year-old lock of hair enabled Rasmussen et al. (2011) to confirm the genomic findings discussed above (Figure 7). Importantly, they were unable to detect any evidence of European admixture and concluded that Indigenous Australians were descendants of that earlier human dispersal into eastern Asia 62–75 kya. Furthermore, these investigators demonstrated that this dispersal was separate from the one that gave rise to modern Asians 25–38 kya and supported the view that present-day Indigenous Australians descended directly from the earliest humans to occupy Australia, thus representing the oldest continuous population outside of Africa. Finally, the data do support multiple dispersal models with separate dispersals of AMH into eastern Asia; that is, the founder group for the Indigenous Australians and the ancestors of most present-day East Asians.

### Spread of Modern Humans: From Great Steppe Nomads to Bronze Age Farmers

The Great Steppe (Mammoth Steppe) stretched from the Atlantic Coast of Europe in the west to Hokkaido in the east. It was the vast steppe ecoregion of Eurasia with extensive temperate grasslands, savannas, and shrublands biome. It supported almost limitless herds of large herbivores such as wild horses, reindeer, musk-ox, and saiga antelopes as well as the woolly mammoth, steppe bison, and woolly rhinoceros. The environment readily supported the hunter-gatherer lifestyle (Oppenheimer, 2003), but it was bitterly cold. By 30 kya, a substantial population of hunter-gatherers was living on and along the Great Steppe. However, by around 18 kya, the Last Glacial Maxim (LGM) began to drive temperatures down even further, causing massive movement of peoples south into Central and Southern Europe taking refuge from the coming of the Ice Age, and adopting a predominantly farming lifestyle by around 5 kya. Agriculture arose independently in at least 11 “centres of origins” scattered over all inhabited continents except Australia from around 11–12 kya (Larson et al., 2014). The benefits of farming over hunter-gathering methods for food triggered the agricultural expansion which spanned over 6,000 years. However, many controversies exist regarding the dispersal process from these regions. The Fertile Crescent (Mesopotamia and Ancient Egypt) in the Levant is considered as one of the earliest “cradle of civilization,” and the early domestication of plants and animals began in the region around 11 kya. Based on craniofacial form studies, it was also one of the earliest “centre of origin,” where the early farming populations migrated westward into Europe and North Africa, northward to Crimea, and also eastward to Mongolia (Brace et al., 2006). Of these, the Neolithic transition from the Near East into Europe that spanned between 9 and 5.5 kya has been the most studied. However, both archeological (Brace et al., 2006) and genetic (Dupanloup et al., 2004) studies have confirmed that the Neolithic and Bronze Age peoples of Europe are not closely related to the modern inhabitants. Nonetheless, these studies supported by mathematical modeling (Fort, 2015) revealed that the transition took place *via* demic and cultural diffusion. The contribution levels of Paleolithic and Neolithic populations to the gene pool of modern European populations were sufficient to support the theory that as the Neolithic populations diffused from the Near East into Europe, they had lived and interbred with the existing hunter-gatherers who had inhabited the regions since Late Pleistocene, and who over time had absorbed the cultures and agricultural way of life of these migrants. Although the dispersion routes from the other “centres of origins” around the globe have not been studied as extensively, it is likely that demic and cultural diffusion had also played an important role in the geographical distribution of modern-day humans, and by association, the distribution of HBV genotypes.

## Theories of HBV Evolution

A number of theories have been proposed for the origin and evolution of HBV, as reviewed by Simmonds (2001). However, the discovery of hepadnaviruses in a number of reptile and amphibian species has radically altered the reasoning behind these theories, revealing deep ancestry across the animal kingdom. The presence of endogenous hepadnavirus species in birds and reptiles suggests an origin >200 mya followed by co-evolution with the host species within the reptile/fish/bird host lineages (Geoghegan et al., 2017; Lauber et al., 2017). The orthohepadnaviruses detected from a number of bat species, which are genetically most related to primate HBV, also support co-evolution of *hepadnaviridae* in the mammalian lineage (Drexler et al., 2013).

When it comes to HBV evolution within the primate lineage, a theory involving co-evolution is unlikely. The lack of distinct evolutionary pathways between human HBV and those from African and Southeast Asian NHP species undermines it (Rasche et al., 2016). Cross-species transmission cases have been reported in multiple studies (Grethe et al., 2000; Hu et al., 2000; Takahashi et al., 2000; Magiorkinis et al., 2005; Simmonds and Midgley, 2005; Dupinay et al., 2013). It is also worth noting that geographical regions, where there are increased opportunities for HBV transmission between human and NHP tend to be areas, where HBV prevalence is high among humans (Southeast Asia and Africa), thus supporting the notion that cross-species transmission may have played an important role in the evolution of modern-day primate HBV genotypes.

Until the discovery of aHBV, it was presumed the modern human HBV genotypes co-evolved and spread with AMH out of Africa (Norder et al., 1994; Magnius and Norder, 1995). However, the finding of five Neolithic and Bronze Age aHBV in Central and Western Europe that could be classified as an extinct genotype of NHP-like aHBV supports a substantially more complex evolutionary history.

Primate HBV is the youngest lineage of the family *Hepadnaviridae*, and its origin is likely to be sometime in the Middle Pleistocene era. Paraskevis et al. had estimated an origin time of 33.6 kya for the primate HBV of humans and Old World NHP species (Paraskevis et al., 2013). However, Yuen et al. had estimated an origin time of 59 kya for the HBV subgenotype C4 alone (Yuen et al., 2019), thus implying the origin time of primate HBV to be substantially older. Such a large discrepancy between the two studies was probably the consequence of different fossil calibration data and HBV sequence datasets used during the phylogenetic inferencing process. Nonetheless, combining the recent findings of aHBV from Krause-Kyora et al. (2018) and Muhlemann et al. (2018) with the knowledge of AMH migration histories have enabled the proposition of an alternative model for the origin of primate HBV. We propose the origin and evolution of modern-day human and Old World NHP HBV could be driven by major AMH migration events, including the early human migration out of Africa during the Upper Paleolithic era (Bergstrom et al., 2021) and the agricultural expansion during the Neolithic (Brace et al., 2006; Larson et al., 2014).

In the scenario, that the unique subgenotype of Indigenous Australians HBV-C4 had originated in Island Southeast Asia and entered Australia around 50 kya, as inferred by Yuen et al. (2019), imply two important events. Firstly, ancestors of the Asian dominant HBV (genotypes B and C) had already been established in Asia during this time period. Secondly, the origin time of Old World primate HBV is substantially older, supporting the theory of an Out of Africa origin. The current geographical distribution of human HBV genotypes also supports the two main routes of human migration out of Africa, with the northern route introducing the ancestral strains of the European dominant HBV into Eurasia (genotypes A and D) and the ancestral strains of Asian dominant HBV introduced along the Indian Ocean coastline into Oceania and Southeast Asia, often referred to as the southern dispersal route. Although the origin of HBV genotypes F and H remains enigmatic, the phylogenetic analysis supports their origin being in the Old World and had diverged from the same common ancestral strain of HBV that gave rise to the present-day human and Old World NHP HBV, and possibly followed by long-term isolation. The long-term isolation event may be associated with the “Beringian standstill” hypothesis (Tamm et al., 2007) that suggests the ancestors of Native Americans had been geographically isolated on the Beringian land bridge for millennia during the past glacial maximum before dispersal into the American continents.

The agricultural revolution that occurred throughout the Neolithic and Bronze Age (Larson et al., 2014) had played an important role in the global dispersion of HBV genotypes. This has been previously noted in phylogeographic studies of genotypes A and D (Kostaki et al., 2018). Prior to this, ancient hunter-gatherer societies were likely to be small and sparsely dispersed. The spread of farming societies during these time periods coincided with population growth worldwide, which in turn enabled the global spread of the ancestral strains of human HBV. The discovery of a potentially extinct genotype most closely related to African NHP HBV in human fossils from the Neolithic and Bronze Age suggests numerous ghost lineages may have existed in the past, and that the landscape of HBV genotypes and subgenotypes in this era was vastly different to today. This is an important fact to take into account during the inferencing process of HBV evolutionary history. The extinct African NHP-like genotype isolates detected in human fossils with ages that spanned over 2,000 years in Central and Western Europe also suggest that it may have been endemic in the region and could have been introduced from the Fertile Crescent, a region with frequent human traffic between Africa and the Levant, as farming cultures diffused from the Near East into Europe (Brace et al., 2006).

The final shaping of the current geographical distribution of HBV genotypes was the result of numerous more specific population-movements post-Bronze Age, rather than by a single major migration event. These include the Medieval Great Migrations in Europe during the first millennium AD, early historical invasions, and slave trades.

## Summary and Conclusion

The recent papers on aHBV from Krause-Kyora et al. (2018) and Muhlemann et al. (2018) provide a unique contribution to the HBV field and novel insights into the origin and dispersal of HBV over the last 7,000 years. These Neolithic and Bronze Age HBV genomes are most closely related to those infecting African NHP, have no close relatives today, possibly went extinct and appear to be generated by recombination. Both papers clearly demonstrate that humans throughout Eurasia have been widely infected with HBV for 1,000 years. Surprisingly, the geographical locations of a number of aHBV genotypes did not match present-day genotypic distribution. For example, genotype A aHBV was identified in South West Russia over 4 kya and the authors propose that it may have descended from an ancient Eurasian tribal group derived from the western Scythian culture, who themselves descended from a Western steppe population (Damgaard et al., 2018), and not from African ancestors as previously thought (Hannoun et al., 2005; Zehender et al., 2015). Interestingly, the three oldest genotype A aHBV strains studied (DA195, RISE386, and RISE387; Muhlemann et al., 2018) lacked the 6 nt insertion characteristic of modern genotype A (Kramvis, 2014) as did the youngest isolate DA119 discovered in this study. These investigators go on to suggest that the ancestors of subgenotypes A1 and A3 would have then been carried *via* migration from Western Eurasia (Pickrell et al., 2014) subsequently, into Africa. The introduction of genotype A into Africa from Western Eurasia is supported by the phylogeographic analysis performed by Kostaki et al. (2018).

All genotype D isolates were found in Central Asia including the ancestral genotype D isolate DA51 from Kyrgyzstan around 2.2 kya supporting the notion of subsequent spread into India and onto the Pacific. The two Neolithic isolates discovered by Krause-Kyora et al. have closely linked to African NHP over 7 kya with no human association. These NHP sequences provided a major contribution to the Neolithic and early Bronze Age aHBV, suggesting an African origin for these extinct genotypes (see Table 1). Finally, Muhlemann et al. (2018) identified ancestral A in isolates RISE386/387, while Datta (2020) further identified chimpanzee and genotype C2 sequences. The ancestors of DA51 with an unknown parent sequence combined to form ancestral genotype A, the oldest known genotype. It is thus hard not to accept the important role of NHP in the evolutionary history of human HBV (Table 1). Modern studies have consistently shown that genotype C is the oldest of the modern HBVs but the data from the aHBV sequences would support a series of successful zoonoses from primates to humans, followed by recombination events with other HBVs, both primate and human, in the 9 genotypes of human HBVs recognizable today.

## Author Contributions

SL: conceptualized and drafted the manuscript. LY: generation of figures. All authors contributed to the article and approved the submitted version.

### Conflict of Interest

The authors declare that the research was conducted in the absence of any commercial or financial relationships that could be construed as a potential conflict of interest.
